# Factors influencing corticomuscular coherence for axial and lower limb musculature in a healthy population: a scoping review

**DOI:** 10.3389/fnhum.2026.1708259

**Published:** 2026-03-11

**Authors:** Nadim Fakhry, Pouya Rabiei, Martine Gagnon, Martin Simoneau, Hugo Massé-Alarie

**Affiliations:** 1 Center for Interdisciplinary Research in Rehabilitation and Social Integration (Cirris), CIUSSS de la Capitale-Nationale, Quebec City, QC, Canada; 2 Faculty of Medicine, Université Laval, Quebec City, QC, Canada; 3 Library, Université Laval, Quebec City, QC, Canada

**Keywords:** axial muscles, corticomuscular coherence, EEG, EMG, healthy population, lower limb, motor control

## Abstract

**Introduction:**

Corticomuscular coherence (CMC) quantifies the frequency-specific coupling between cortical and muscular activity and is increasingly used to probe motor-control mechanisms. However, the factors that consistently influence CMC in axial and lower-limb muscles remain unclear.

**Objective:**

The objective of this study is to map and critically describe experimental factors and methodological choices that have been studied and their potential influence on CMC of axial and lower limb muscles measured in healthy humans.

**Methods:**

A scoping review was conducted following PRISMA-ScR guidelines. CINAHL, MEDLINE (Ovid), Embase, and Web of Science were searched from the date of inception to March 5th, 2024. Eligible studies that (i) computed CMC, (ii) recorded cortical activity with EEG or MEG, (iii) analyzed trunk or lower limb EMG, and (iv) compared CMC across experimental conditions or participant groups were included. Two reviewers independently screened records, extracted demographic, neurophysiological, task, and signal-processing variables, and grouped studies by the factor examined; third reviewer resolved discrepancies. Results were synthesized descriptively.

**Results:**

Four factors showed the most consistent influence on CMC: (1) Age: younger adults exhibit higher CMC than older adults (2) Muscle-specificity: the tibialis anterior (TA) displays stronger CMC than other axial or lower-limb muscles; (3) Contraction type: isotonic and eccentric/quasi-isotonic contractions elicit greater CMC than isometric contractions; (4) Athletic status: non-athletes demonstrate higher CMC than trained individuals. The effects of fatigue, contraction intensity, posture, or walking tasks were inconsistent. Methodologically, most studies employed EEG (single Cz channel) and rectified EMG; MEG, source localization, and longitudinal approaches were seldom used.

**Discussion:**

Current evidence indicates that participant characteristics (age, athletic status) and task parameters (muscle tested, contraction type) can impact CMC, but heterogeneity in study design and analysis hampers direct comparison and causal inference. Future research should adopt longitudinal designs, standardized protocols, and advanced source localization techniques to clarify the mechanisms governing CMC in axial and lower-limb musculature.

## 1 Introduction

Corticomuscular coherence (CMC) between electroencephalography (EEG), magnetoencephalography (MEG), or local field potentials and electromyography (EMG) is used to understand the cortical control of movement (Mima and Hallett, 1999). Coherence, defined as the mathematical correlation between spatially distinct neural systems, is commonly interpreted as an index of functional coupling or communication between these systems (Bressler et al., 1993; Classen et al., 1998; Roelfsema et al., 1997). CMC is an extension of the Pearson correlation coefficient in the frequency domain (Mima and Hallett, 1999) that informs on the strength of the linear relationship between two signals (Grosse et al., 2003). Moreover, it has been suggested that CMC represents the reciprocal communication between the sensorimotor cortex and contracting muscles through descending and ascending pathways (Kilner et al., 2004; Mima et al., 2000; Ohara et al., 2000; Pohja et al., 2002; Riddle and Baker, 2005). CMC is measured in different frequency bands; each band is thought to provide insight into different physiological mechanisms of neural control. The most studied frequency range is the *β*-band (12–30 Hz) for which significant CMC is consistently detected (Engel and Fries, 2010; Mehrkanoon et al., 2014; Mima et al., 2002). β-band CMC has been suggested to represent a fundamental component of sensorimotor integration, highlighting the coordination between cortical and muscular activity during movement (Peng et al., 2024). Other frequency bands such as *α* and *γ*-band are also frequently measured in CMC studies (Jensen et al., 2018; Ozdemir et al., 2018; Vecchio et al., 2008; Yoshida et al., 2017a). It has been suggested that coherence at higher frequencies (γ-band) could indicate the integration of multisensory inputs contributing to motor planning (Omlor et al., 2007). However, the functional significance of α-band CMC remains poorly understood, and further research is needed to clarify its role. The functional significance of these bands helps to better understand neural processing contributing to a specific motor task.

Conway et al. (1995) conducted the first study on the CMC utilizing axial gradiometer magnetoencephalography (MEG) and surface EMG recordings from the first dorsal interosseous muscles (FDI). Significant CMC, primarily in the *β*-band were observed exclusively on the sensorimotor cortex contralateral to the contracting muscle in all healthy participants (Conway et al., 1995). Most research has primarily measured CMC of upper limbs (Chakarov et al., 2009; Halliday et al., 1998; Kristeva-Feige et al., 2002; Mima et al., 2000; Omlor et al., 2007; Yang et al., 2009), likely due to the stronger CMC reported for upper limbs compared to lower limbs and axial muscles (Murayama et al., 2001). This stronger CMC may be explained by the abundance of corticospinal projections to *α*-motor neurons of the upper limbs, especially the hand, compared to lower limb and axial muscles (Ghez and Krakauer, 2000; Lemon, 2008). Lower limb and axial muscles contribute to postural and gait control (Bogduk et al., 1992; Kumai et al., 2022). Multiple neural areas such as the sensorimotor cortex, brainstem nuclei, and some cerebellum areas, contribute to postural control (Takakusaki, 2017). Despite postural muscles exhibiting weaker corticospinal connectivity than upper limb muscles, there is evidence of corticospinal control of these muscles in humans (Desmons et al., 2024; Desmons et al., 2023). Therefore, CMC can help determine the contribution of sensorimotor cortical areas to the control of lower limb and axial muscles.

While an increasing number of CMC studies have been published in recent years, no study to date has comprehensively identified the factors influencing CMC in axial and lower limb muscles in healthy individuals, which may differ from those in upper limb muscles. Some studies have investigated specific characteristics, such as age (Bayram et al., 2015; James et al., 2008; Johnson and Shinohara, 2012), fatigue (McManus et al., 2016; Yang et al., 2009), and reported significant effects of these factors for CMC of hand and forearm muscles. Nonetheless, it is still unknown whether the factors influencing CMC in the upper limb are the same as those in the lower limb and axial muscles. Moreover, although the CMC of *β*-band is the most studied, many studies also compute CMC of other bands. It remains unclear if they can discriminate between different motor tasks or conditions. Building on this work, this scoping review aims to conduct an in-depth literature analysis to identify, analyze, and synthesize the methodology used to compute CMC and the factors studied that potentially influence CMC, focusing on axial and lower limb muscles in healthy individuals.

## 2 Methods

### 2.1 Data source and search

Our review was performed according to the recommendations for a scoping review (PRISMA-ScR) (Tricco et al., 2018). Four databases underwent an electronic bibliographical search: CINHAL, Medline (OVID), Embase, and Web of Science, from the date of inception to March 5th, 2024. The search was updated in September 2025. There was no constraint on the publication year. The literature search strategies were developed based on three concepts: (1) imaging techniques to record cortical activity, (2) techniques to measure muscular activity, and (3) corticomuscular coherence (full search strategies are provided in Supplementary material). These concepts were selected because they are the fundamental components required to assess the relationship between cortical and muscular activity through the lens of CMC. This strategy guarantees a comprehensive exploration of studies combining these methodologies to answer relevant neurophysiology and motor control questions.

### 2.2 Eligibility criteria

#### 2.2.1 Inclusion criteria

Studies were included if:

i CMC was calculated;
ii cortical activity was measured by electroencephalography (EEG) or magnetoencephalography (MEG);
iii activity of the trunk or lower limb muscles was measured by any type of electromyography (EMG - e.g., surface, intramuscular, high density);
iv CMC from two experimental conditions were compared (e.g., level of muscle contraction, type of movement [isometric vs. isotonic], different muscles);

OR CMC was measured before and after the manipulation of physiological independent variables (e.g., pre- and post-fatigue or pre- and post- neurostimulation);
OR CMC of a given muscle was compared between groups of participants having distinct individual characteristics (e.g., different age groups [younger vs. older], athletes vs. non-athletes);

v Healthy participants were tested;
vi Only studies published in English or French were included.

#### 2.2.2 Exclusion criteria

Studies were excluded when they met the following criteria:

- CMC of upper limb muscles was measured exclusively (i.e., only CMC of upper limb muscles was computed). It is important to note that if CMC of upper limb muscles was compared to lower limb or axial muscles, the study was included;
- Cortical activity was measured by electrocorticography (ECoG) due to its invasiveness;
- CMC was calculated in participants living with a disease/health condition (e.g., stroke, pain, Parkinson’s);
- Inter/intra-muscular coherence was computed instead of CMC;
- Systematic literature review, case study, conference, poster presentation.

Studies computing CMC exclusively of the upper limb were excluded because (i) they increase the heterogeneity and number of the factors that may have been studied, (ii) they increase heterogeneity of pooled studies due to potential differences in neural processing, (iii) the body of literature available was too numerous and too diverse for a single scoping review.

### 2.3 Study selection

The Covidence program (Veritas Health Innovation, Melbourne, Australia) was used to import all the records from the databases found by the literature search. Two independent reviewers (NF, PR) screened the abstracts and titles using the predefined eligibility criteria after duplicates were eliminated from Covidence. Articles that potentially met the eligibility criteria were independently reviewed (full-text screening) by two authors (NF, PR). At each stage of the scoping review, a third reviewer (HMA) was brought in to resolve disagreements through open discussion and consensus.

### 2.4 Data extraction and synthesis

One author (NF) extracted data from the included studies: demographic characteristics of the studied samples (e.g., sex, age). We also retrieved the parameters used to record cortical signals: brain technique (MEG or EEG), number of EEG electrodes used to calculate the corticomuscular coherence, and EEG channels analyzed for coherence. Moreover, whether the choice of EEG channel was explained, source analysis conducted (yes or no, with specifics if applicable), type of coherence measured (static or dynamic), and the frequency bands examined were noted. Muscle contraction details were extracted, including the level of contraction, type of contraction (form and duration), and task description. The targeted muscles were identified, and whether rectified EMG was used (yes or no, with explanation) to calculate CMC. Finally, the objectives of the articles with the conditions compared were extracted, along with the results and statistics related to CMC.

To address the objectives of the scoping review, articles testing similar factors or individual characteristics related to CMC were gathered. Each group of studies was named based on the representative factor/characteristics studied (e.g., age) and described altogether. Finally, results of each factor/characteristic on CMC were reported descriptively. When possible, we tried to reduce heterogeneity by pooling the results of a potential factor using the same muscle and the same band. For example, if age groups were compared, we pooled the results for the same muscle (e.g., tibialis anterior) and a same band (e.g., *β*-band). Considering the objective of the study and the heterogeneity of the included studies, we did not attempt to conduct meta-analyses.

Importantly, only frequency-domain correlation coefficients were extracted to quantify CMC across the different frequency bands. Other outcomes that may inform different neurophysiological domains were neither extracted nor synthesized in the present review.

## 3 Results

### 3.1 Characteristics of included studies

**Figure 1:**
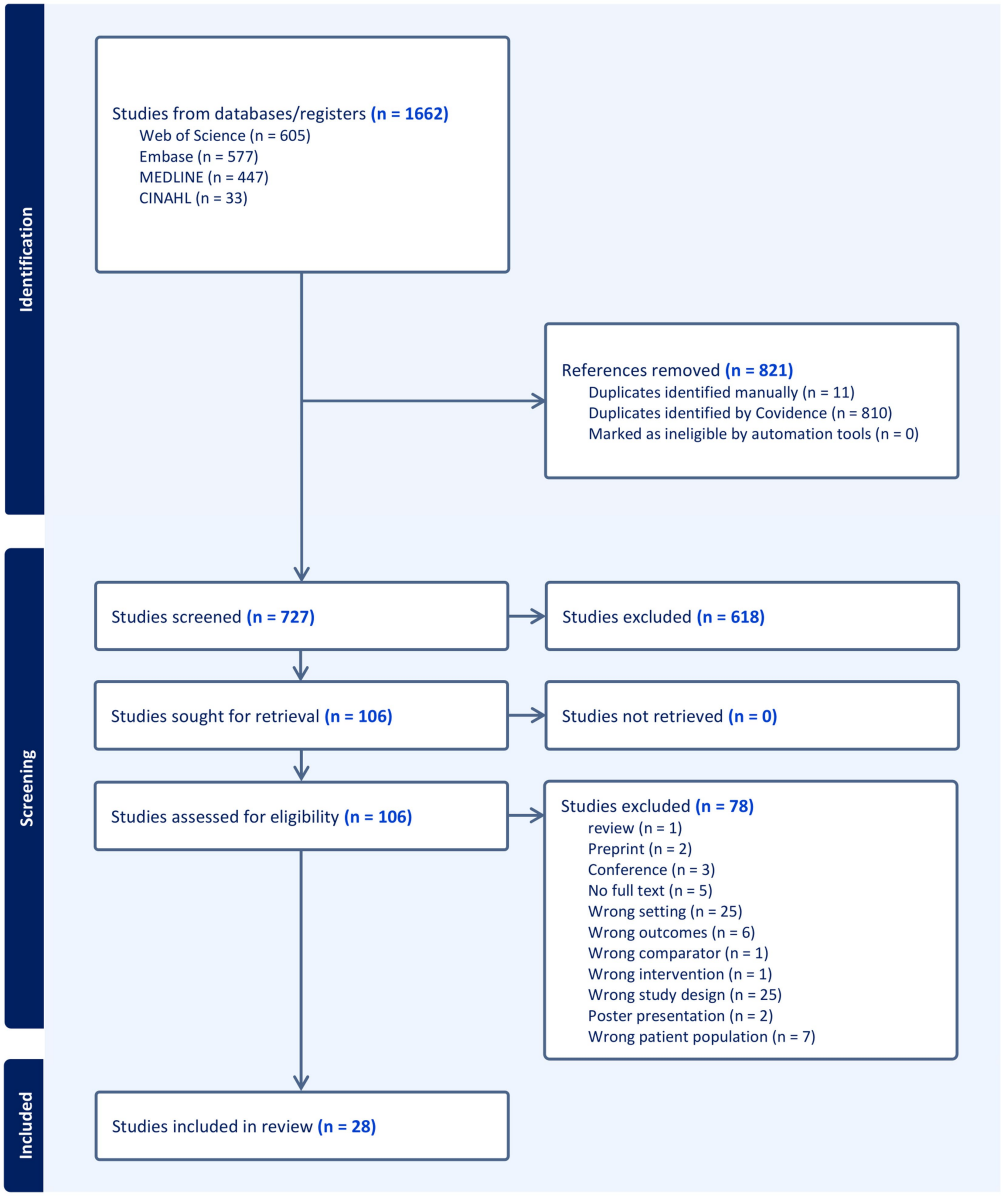
PRISMA flow chart of the scoping review.

Figure 1 presents the PRISMA flowchart and summarizes the outputs from each selection process step. From the 727 articles screened, 28 studies measuring CMC for axial and lower limb muscles in a healthy population were included in this review (Figure 1).

### 3.2 Characteristics of the population in included studies

Table 1 reports the characteristics of the sample in the included studies. Briefly, the median number of participants recruited in each study was 15 [min-max: 7–60], indicating a small sample size with considerable variability between studies. When reported, the mean age of the participants included in the studies ranges between 22 and 28 years, except when older adults were studied. The mean age of older participants from individual studies ranges between 66 and 81 years. The median percentage of females in included studies was 33.3 [0–100] %.

**Table 1:** Characteristics of the study samples in the included studies.

References	Groups (n)	Age [years (SD)*]	% Female
Correia et al. (2024)	19	26 (6)	0
Dal Maso et al. (2017)	10 ST 11 ET	ST: 24 (4) ET: 22 (2)	0 0
Elie et al. (2021)	13	28 (7)	0
Gennaro and de Bruin (2020)	9 Young 9 Old	26 (3) 73 (6)	55.6 33.3
Glories et al. (2021)	25	26 (6)	n.a.
Gwin and Ferris (2012)	8	Between 21–31	12.5
Ushijima et al. (2017)	11	Between 21–29	0
Jacobs et al. (2015)	10	23	50
Jensen et al. (2018)	16	23 (5)	62.5
Kenville et al. (2020)	11	28 (5)	0
Liang et al. (2022)	15	24 (2)	13.3
Masakado et al. (2008)	15	Between 20–47	57.1
Masakado and Nielsen (2008)	9	Between 22–28	n.a.
Murayama et al. (2001)	8	Between 24–39	37.5
Murnaghan et al. (2014)	12	24 (4)	66.7
Ozdemir et al. (2018)	10 Young 9 Older	26 (3) 81 (6)	40 66.7
Perez et al. (2006)	11	27 (4)	63.6
Poortvliet et al. (2015)	17	33 (6)	17.7
Roeder et al. (2018)	24	26 (3)	50
Spedden et al. (2019) and Spedden et al. (2018)	15 Young 15 Older	22 (1) 68 (3)	53.3 53.3
Masakado et al. (2011)	7	23 (2)	0
Ushiyama et al. (2010)	24 untrained 12 skill-trained 10 strength-trained	Between 21–31 Between 19–29 Between 19–22	50 100 0
Ushiyama et al. (2012)	11	Between 22–31	27.3
Vecchio et al. (2008)	19 karate athletes 14 karate amateurs 9 nonathletes 18 elite fencing	Between 18–32 Between 23–43 Between 23–43 Between 19–36	47.4 28.6 33.3 66.7
Yoshida et al. (2017b)	15	27 (7)	0
Yoshida et al. (2017a)	15 young 9 old	27 (7) 66 (7)	0 0
Zaback et al. (2022)	28	24 (5)	39.3

*Measure of the variability is SD unless otherwise specified; ST, strength training; ET, endurance training; n.a., not available.

### 3.3 Methods used to compute CMC

**Figure 2:**
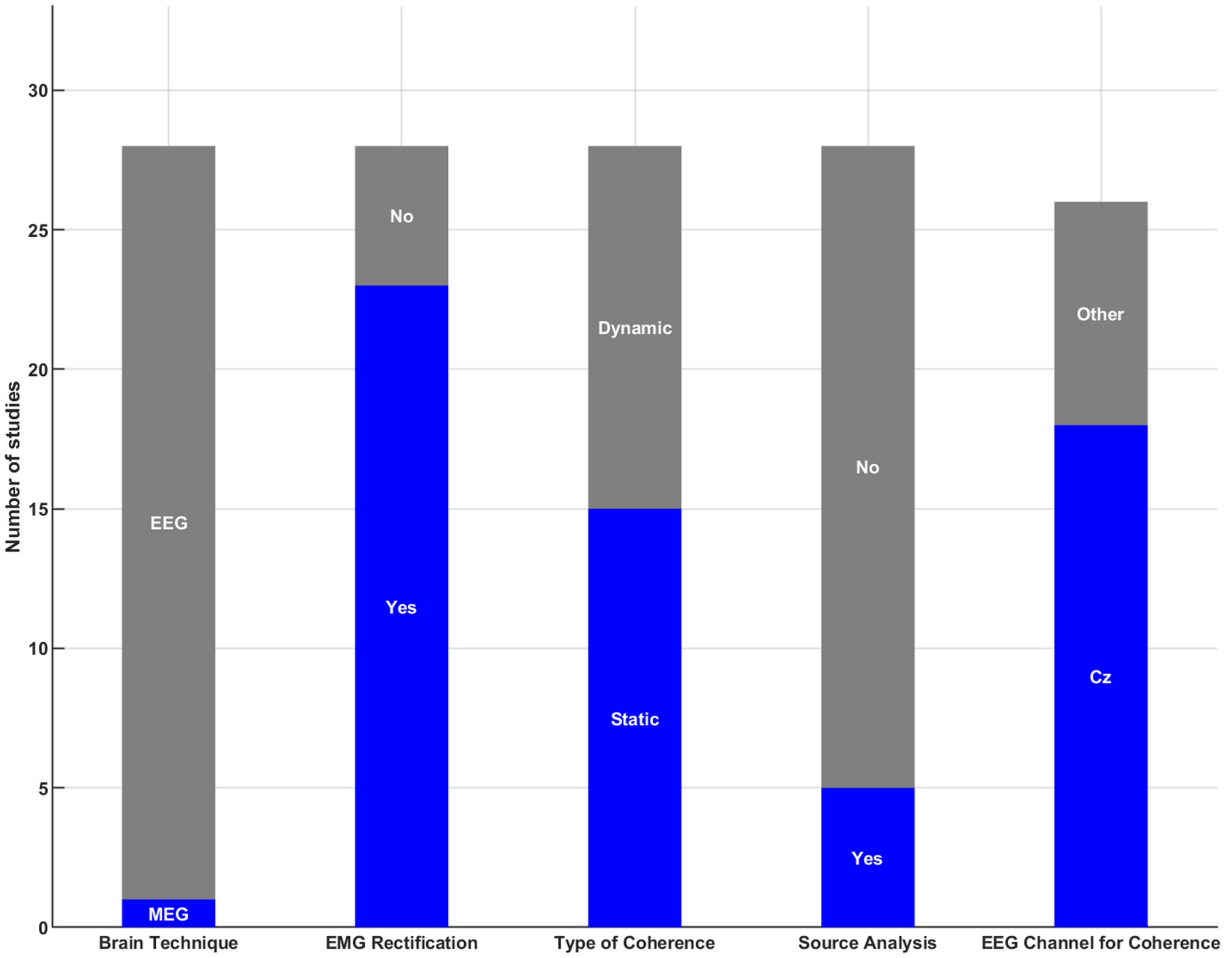
Overview of signal acquisition and processing methods for CMC analysis.

The CMC can be computed using various methodologies regarding data collection (e.g., brain technique) and data processing (e.g., EMG rectification, source analysis). Figure 2 illustrates the methods employed by the included studies to collect signals and calculate CMC. Regarding brain techniques, all studies used EEG (27 out of 28) except one which used MEG. All studies used surface EMG. Concerning EMG rectification, 23 out of 28 studies rectified the EMG signals before computing CMC. Static CMC (single window analysis) was used in more studies than dynamic CMC (time-varying analysis) (static: *n* = 15; dynamic: *n* = 13). Only 5 out of 28 studies used source analysis to compute CMC. Most studies used the electrode located at Cz to calculate CMC for lower limb and axial muscles (*n* = 18). In contrast, other studies selected a single or a bundle of electrodes in the sensorimotor cortex. For example, Correia et al. (2024) used the C3 electrode, and (Poortvliet et al., 2015) used a cluster of electrodes from the sensorimotor cortex (i.e., electrodes 30, 31, 52, 54).

### 3.4 Factors studied potentially impacting corticomuscular coherence

**Figure 3:**
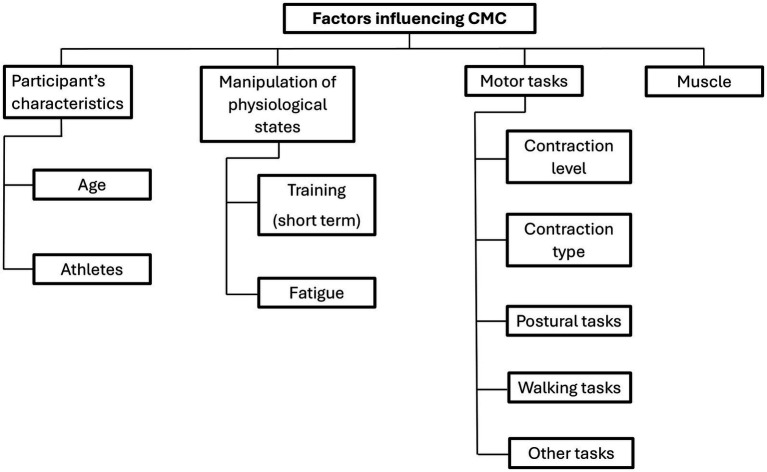
Factors studied potentially influencing corticomuscular coherence (CMC).

The factors or characteristics studied that have been hypothesized to influence CMC potentially were pooled into four main categories: (1) participant characteristics, specifically age and athletic status; (2) manipulation of physiological states, including short-term training and fatigue; (3) motor task-related factors, such as contraction level, and contraction type, postural tasks, walking tasks, other tasks; and (4) muscle (Figure 3).

#### 3.4.1 Participant characteristics

Participant characteristics included studies that compared CMC for groups of participants having different characteristics, such as being of different ages or being an athlete (vs. non-athlete). Methods used and results are reported in Table 2.

**Table 2:** Methodological factors and results of CMC for participant characteristics.

Reference	Band frequency	Muscle	Task	CMC results
Age				
Gennaro and de Bruin (2020)	β and lower *γ*	TA	Overground walking	NSS
Ozdemir et al. (2018)	δ, θ, *α*, *β* and γ	TA and RF	Quiet stance postural tasks and postural perturbations	α: young > old for TA β: young < old for RF
Spedden et al. (2018)	α, β	TA, SOL, and GM	Isometric dorsiflexion and plantar flexion	β: young > old for TA and GM
Spedden et al. (2019)	β and γ	TA	Normal and visually guided gait	β: young > old for TA γ: young > old for TA
Yoshida et al. (2017a)	δ, θ, α, β and γ	TA and GM	Cyclical, anti-phasic ankle movements	β: young > old for TA and GM
Athletes				
Vecchio et al. (2008)	α, β and γ	TA, GL, and EO	Quiet upright standing	α: nonathletes > karate amateurs and elite karate athletes for GL
Ushiyama et al. (2010)	β	SOL, TA, BF, and RF	Tonic isometric contractions	β: untrained > Ballet dancers and weightlifters for SOL, TA and BF
Dal Maso et al. (2017)	β	VM, RF, BF, and GAS	Isometric knee contractions	β: strength-trained > endurance-trained for VM, RF, BF and GAS

NSS, not statistically significant; TA, tibilalis anterior; GM, gastrocnemius medialis; RF, rectus femoris; BF, biceps femoris; SOL, soleus; EO, external oblique; GL, gastrocnemius lateralis; VM, vastus medialis; GAS, gastrocnemius.

##### 3.4.1.1 Age

Table 3 pooled the results to foster the interpretation of the results. Many studies have detected significant effects of age. For example, the CMC of the TA muscle was investigated in five different studies. The CMC in the *α*-band was higher in young adults compared to older adults in one study (Ozdemir et al., 2018), but not significantly different in the two other studies (Spedden et al., 2019; Yoshida et al., 2017a). For the *β*-band, three studies (Spedden et al., 2019; Spedden et al., 2018; Yoshida et al., 2017a) reported higher CMC for young adults, whereas no difference was present in two studies (Gennaro and de Bruin, 2020; Ozdemir et al., 2018). For the *γ*-band, one study (Spedden et al., 2019) observed higher TA CMC in young compared to older adults. No study reported significant differences in *δ* and *θ* frequencies. For the gastrocnemius medialis (GM) muscle, 3 studies compared young and older adults. Two studies (Spedden et al., 2018; Yoshida et al., 2017a) observed a higher GM *β*-band in young compared to older adults and one study (Ozdemir et al., 2018) reported no difference (Table 3). Interestingly, none of the other frequency bands compared showed significant differences among young and older adults.

**Table 3:** Summary of pooled findings highlighting age-related effects on CMC.

Muscles	Bands	Gennaro and de Bruin (2020)	Ozdemir et al. (2018)	Spedden et al. (2018)	Spedden et al. (2019)	Yoshida et al. (2017a)	% young > old
TA	δ	-	≠	-	-	≠	0%
	θ	-	≠	-	-	≠	0%
	α	-	↑	≠	-	≠	33%
	β	≠	≠	↑	↑	↑	60%
	γ	≠	≠	-	↑	≠	25%
GM	δ	-	-	-	-	≠	0%
	θ	-	-	-	-	≠	0%
	α	-	-	≠	-	≠	0%
	β	-	-	↑	-	↑	100%
	γ	-	-	-	-	≠	0%

##### 3.4.1.2 Athletes

Two studies reported that CMC was greater in non-athletes than in athletes, but in different frequency bands depending on the study (α: Vecchio et al., 2008; *β*: Ushiyama et al., 2010; Table 2). Additionally, one study found that strength-trained athletes exhibited higher *β*-band CMC than endurance-trained athletes for multiple lower limb muscles (Dal Maso et al., 2017).

#### 3.4.2 Manipulation of physiological states

The manipulation of physiological states included studies that compared the CMC before and after an intervention (e.g., short-term training or a fatigue task). The methods used and the corresponding results are presented in Table 4.

**Table 4:** Methodological factors and results of CMC for manipulation of physiological states.

Reference	Band frequency	Muscle	Intervention	CMC results
Training				
Elie et al. (2021)	α and β	TA, GL, SOL, and GM	Strength training for 3 weeks	NSS
Perez et al. (2006)	β	TA	Single session visuo-motor skill learning	β**: ↑** post-training
Fatigue				
Correia et al. (2024)	δ, θ, α, β and γ	BF, RF, STN, VL and VM	Prone, unloaded knee flexion/extension at maximum voluntary rate over eight blocks of 10 s with 5 s of rest between blocks	θ: ↓ post- fatigue for BF, RF, STN, VL and VM
Masakado et al. (2011)	β	TA	Sustained isometric dorsiflexion at 50% of MVC until exhaustion	β: **↑** post-fatigue for TA
Ushijima et al. (2017)	β and γ	TA	Randomized one-minute foot flexing contractions at 10, 15, and 30% MVC	β: **↑** significantly over time

NSS, not statistically significant; TA, tibilalis anterior; GM, gastrocnemius medialis; RF, rectus femoris; SOL, soleus; GL, gastrocnemius lateralis; VM, vastus medialis; VL, vastus lateralis; IO, internal oblique; LES, Lumbar erector spinae; TES, thoracic erector spinae; STN, semitendinosus; MVC, maximal voluntary contraction.

##### 3.4.2.1 Training

We identified two studies that aimed to examine the effect of training. The first one examined the effect of a 3-week strength training program on CMC but did not report any significant difference (Elie et al., 2021). In contrast, Perez et al. (2006) found an increase in the *β*-band CMC for the TA after a visuomotor training consisting of tracking a line that varied randomly in time by moving the ankle.

##### 3.4.2.2 Fatigue

Regarding the effect of fatigue, two included studies reported conflicting results, although the same muscles were not studied. Correia et al. (2024) found that CMC was reduced after the fatiguing task in the *θ*-band for multiple lower limb muscles, whereas 2 studies reported an augmentation of the *β*-band CMC for the TA following fatigue (Masakado et al., 2011; Ushijima et al., 2017; Table 4).

#### 3.4.3 Motor tasks

The “motor tasks” factor refers to the comparison of CMC between different motor tasks. It may refer to the comparison of the CMC resulting from different contraction levels or contraction types within the same muscle. Additionally, we included two sub-categories comparing CMC across postural and walking tasks (Table 5).

**Table 5:** Methodological factors and results of CMC for motor task.

Reference	Band frequency	Muscle	Task	CMC results
Contraction level				
Dal Maso et al. (2017)	β	VM, RF, BF and GAS	Isometric knee contractions at 20, 40, 60, and 80% MVC	β: ↓ more in antagonist than in agonist muscles, as torque level **↑**
Ushiyama et al. (2012)	β and γ	TA and SOL	Tonic isometric voluntary dorsiflexion/plantar flexion at 10, 20, 30, 40, 50, 60, and 70% MVC	CMC transitioned from β to γ band with increasing contraction level for TA
Contraction types				
Gwin and Ferris (2012)	β and γ	TA, SOL, VL, VM, GM, GL, MH and RF	Isometric and isotonic movements (concentric followed by eccentric)	γ: isotonic > isometric for TA, SOL, VL, GM, MH, RF
Kenville et al. (2020)	β and γ	VL, VM, TA, ES	Squats, divided into three successive movement periods (eccentric, isometric and concentric)	β: eccentric and concentric > isometric γ: eccentric > isometric for all muscles
Masakado and Nielsen (2008)	β	TA	Task 1: isometric ramp and hold contraction Task 2: quasi-isotonic ramp and hold contraction or slow quasi-isotonic contraction	β: quasi-isotonic > isometric for TA
Glories et al. (2021)	β and γ	SOL and GM	Plantar flexion	β: isometric> eccentric for SOL β: NSS for GM γ: isometric> eccentric and concentric for SOL and GM
Postural tasks				
Jacobs et al. (2015)	β	TA and GL	Effects of stance width, vision, and surface compliance during human standing balance	β: Wide stance > narrow-stance condition eyes open for GL
Liang et al. (2022)	θ, α, β, and γ	TA, SOL, GAS, VM and BF	Task 1: bipedal standing Task 2: unipodal stance Task 3: unipodal stance on a foam platform	θ**: ↑** with task difficulty for all muscles
Masakado et al. (2008)	θ, α, β, and γ	SOL	Task 1: tonic contraction in the sitting position (control task) Task 2: standing still Task 3: standing on one foot Task 4: standing, bending forward Task 5: stamping the ground by taking out the right foot slightly before the left one	β: Significant → tonic contraction and stamping the ground for SOL
Murnaghan et al. (2014)	β	GAS and SOL	Participants stood as still as possible in an apparatus that either allowed (1) free movement of the center of mass (“Unlocked”) or (2) impeded it (“Locked”)	NSS
Zaback et al. (2022)	β and γ	SOL	Low and high height-related postural threats	γ: ↑when exposed to high threats
Walking tasks				
Roeder et al. (2018)	θ, α, β and γ	TA	Overground and treadmill walking	β: overground walking> treadmill walking for TA
Jensen et al. (2018)	α, β, and γ	SOL, GM, and TA	Normal walking and visually guided walking.	NSS
Different tasks				
Yoshida et al. (2017b)	β	TA and GM	Self-paced and externally paced cyclical ankle (with metronome)	NSS
Poortvliet et al. (2015)	β and γ	RF, VL, VM, STN and BF	Task 1: Force-control of knee extension Task 2: Position control of knee angle (loaded)	NSS

NSS, not statistically significant; TA, tibialis anterior; GM, gastrocnemius medialis; RF, rectus femoris; BF, biceps femoris; SOL, soleus; EO, external oblique; GL, gastrocnemius lateralis; VM, vastus medialis; VL, vastus lateralis; GAS, gastrocnemius; STN, semitendinosus; ES, erector spinae; MH, medial hamstring; MVC, maximal voluntary contraction.

##### 3.4.3.1 Contraction level

Regarding the contraction level, Dal Maso et al. (2017) showed that the *β*-band CMC decreases more in antagonist than in agonist muscles as the torque level increases. Additionally, Ushiyama et al. (2012) found that CMC transitions from the β to the *γ* band with increasing contraction levels in the TA muscle (Table 5).

##### 3.4.3.2 Contraction types

In terms of contraction types, studies reported that CMC is consistently lower in isometric contractions compared to other types of contractions (e.g., isotonic [concentric, excentric] - (Gwin and Ferris, 2012; Kenville et al., 2020; Masakado and Nielsen, 2008)) as described in Table 5. Specifically, CMC in the *β* and γ bands, for the TA muscle, was lower in isometric contraction compared to different types of isotonic contraction in 2 out of 3 studies. Similarly, CMC of the vastus lateralis (VL) was lower in the isometric task compared to isotonic in 1 (Kenville et al., 2020) out of 2 studies (Gwin and Ferris, 2012) in the β band, and in 2 (Gwin and Ferris, 2012; Kenville et al., 2020) out of 2 studies in the γ-band. For the vastus medialis (VM), β and γ bands CMC was lower for isometric contraction in one (Kenville et al., 2020) out of two studies (Gwin and Ferris, 2012).

##### 3.4.3.3 Postural tasks

Considering the heterogeneity of the tasks compared, it was impossible to pool them based on similar outcomes measured (muscle, band, etc.). No overall trend was observed. Although some studies reported that more difficult postural tasks increase CMC [regardless of the bands studied (Liang et al., 2022), others did not (Jacobs et al., 2015; Masakado et al., 2008)]. For example, Jacobs et al. (2015) reported that the β-band CMC in the gastrocnemius lateralis muscle was greater in a wide stance than a narrow stance, under eyes-open conditions.

##### 3.4.3.4 Walking tasks

Two studies tested CMC during walking tasks. Roeder et al. (2018) reported greater β-band CMC in the TA muscle during overground walking than treadmill walking. Conversely, Jensen et al. (2018) found no significant difference between normal and visually guided walking.

##### 3.4.3.5 Other tasks

In this section, we grouped studies that compared two different tasks that did not correspond to the other subcategories. None of the two studies found significant differences between the tasks (Poortvliet et al., 2015; Yoshida et al., 2017b) (see Table 5 for the detail of motor tasks compared).

#### 3.4.4 Muscle type

Two studies compared CMC across different muscles. The first study found that the β-band CMC was higher in the TA and FDI muscles compared to the paraspinal and abdominal muscles (Murayama et al., 2001). Conversely, in a study led by Ushiyama et al. (2010), higher β-band CMC was observed in distal lower limb muscles like the soleus (SOL) and TA muscles, compared to the upper limb (wrist and arm muscles) and other more proximal lower limb muscles (Table 6).

**Table 6:** Methodological factors and results of CMC for muscle type.

Reference	Band frequency	Muscles	Task	CMC results
Murayama et al. (2001)	β	PS, FDI, ABD, and TA	Isometric contractions	β: TA & FDI > PS & ABD
Ushiyama et al. (2010)	β	FDI, FCR, ECR, BB, TB, SOL, TA, BF, and RF	Tonic isometric contractions	β: TA & SOL > FDI FCR, ECR, BB, TB, BF & RF

TA, tibilalis anterior; PS, paraspinal; ABD, abdominal; FDI, first dorsal interosseus; FCR, flexor carpi radialis; ECR, extensor carpi radialis; BB, biceps brachii; TB, triceps brachii; BF, biceps femoris; RF, rectus femoris.

## 4 Discussion

This scoping review aimed to map the existing literature on factors studied that potentially influence CMC in axial and lower limb muscles. Our findings highlight several important trends and gaps in the current body of research. Specifically, the main factors studied include: (1) participant characteristics, (2) manipulation of physiological states, (3) motor task-related factors, and (4) muscle. More specifically, age, type of contraction, athletic status, and muscle factors consistently influenced the level of CMC. For the motor tasks, the heterogeneity of the studies precludes any formal interpretation of the results. Although these results are mainly based on cross-sectional studies, precluding strong causal effects of these factors on CMC, we will discuss the potential underlying mechanisms in the following sections.

### 4.1 Potential effect of age on CMC

One of the most critical factors influencing CMC consistently is age. Indeed, CMC is often higher in young participants compared to older adults. Physiological changes occurring during aging could explain these results. Indeed, a quantitative study of myelinated fibers in the lateral corticospinal tract was conducted by Terao et al. (1994) at the C6, T7, and L4 spinal levels in 20 individuals aged 19 to 90 years, all of whom had died from non-neurological causes. The results revealed a significant age-related decline in the density of small myelinated fibers (Terao et al., 1994). In the motor cortex, age-related degenerative changes have been reported in the dendrites of 75–80% of Betz cells - whose axons constitute a part of the corticospinal tract - in older adults. In contrast, the dendritic changes in smaller pyramidal neurons appear less pronounced (Scheibel et al., 1977). In addition, spine density of apical dendrites in primary motor cortex (M1), collaterals of dendrites (Nakamura et al., 1985; Watanabe, 1981), and dendritic arborization (Anderson and Rutledge, 1996) were found to decrease progressively with age. Although aging does not seem to cause a significant loss of neurons in the M1 (Haug and Eggers, 1991), age-related deterioration of white matter microstructure is likely to impair corticospinal connectivity (Sala et al., 2012; Salat et al., 2005). The latter findings indicate that multiple changes within the sensorimotor cortex and the corticospinal tract occur with aging and can explain the decrease in CMC. In addition to these degenerative changes in motor areas, neural processing of motor control seems to involve more widespread brain areas in older compared to young adults (Seidler et al., 2010). Thus, CMC measured using electrodes over sensorimotor areas may reflect this change in neural processing in aging. Altogether, these findings indicate that multiple changes within the sensorimotor cortex, the corticospinal tract, and the neural processing of motor control occur with aging and may explain the decrease in CMC.

### 4.2 Potential effect of athletic status on CMC

Non-trained participants (i.e., normoactive individuals) exhibited higher CMC compared to those who train regularly or are athletes. Whether innate or experience-dependent, the origins of inter-individual variability in CMC remain unclear (Ushiyama et al., 2010). In the context of training-induced neural adaptations, Semmler et al. (2004) investigated motor unit coherence and observed that the coherence strength between motor unit pairs in the FDI muscle, particularly within the 10–30 Hz range, was lower in the right (dominant) hand compared to the left in untrained right-handed individuals. This coherence was even further reduced in both hands of trained musicians. These findings, although based on motor unit coherence and from upper limb studies suggest that prolonged and specialized muscle use may diminish the influence of common oscillatory inputs, mainly from the sensorimotor cortex, during sustained contractions (Semmler et al., 2004). This is similar to our included studies where trained athletes [e.g., karate (Vecchio et al., 2008)], ballet dancers, and weightlifters (Ushiyama et al., 2010) demonstrated lower CMC compared to untrained athletes. This could represent the automatization of sensorimotor control in athletes, with wider cortical networks or enhanced contribution of subcortical areas. It is important to consider that the results discussed come from the upper limb and may not translate to the lower limb and axial muscles.

### 4.3 Potential effect of muscle on CMC

The findings also showed that lower limb muscles, specifically the TA muscle, which is controlled by neurons situated deep within the cerebral sulcus, exhibited the highest CMC compared to other studied muscles. Ushiyama et al. (2010) suggest that, despite the depth of these cortical neurons within the longitudinal fissure, EEG electrodes over Cz were still effective for capturing motor-related activity and calculating CMC (Ushiyama et al., 2010). In fact, movement-related cortical potentials have been consistently identified in EEG recordings over the M1, not only during voluntary hand movements (Shibasaki et al., 1980; Terada et al., 1995), but also during foot movements (Shibasaki et al., 1981; Terada et al., 1999). Using transcranial magnetic stimulation, researchers have provided evidence that TA motoneurons showed almost exclusive facilitation and little variability in response amplitude. In comparison, the excitability of corticospinal projections on SOL and medial gastrocnemius (MG) motoneurons was significantly weaker (Brouwer and Qiao, 1995). One study even demonstrated that the TA muscle’s CMC was higher than that of upper limb muscles, including finger and wrist muscles (Ushiyama et al., 2010). This is surprising because the upper limb muscles are typically involved in more precise and dexterous tasks and thus are generally thought to have stronger corticospinal control (Baker et al., 1997). Nonetheless, another study reported similar CMC between TA and FDI (Murayama et al., 2001). It is possible than methodological aspects could explain this discrepancy (e.g., channels used, source analysis – see section on Methodological factors for in-depth discussion).

### 4.4 Potential effect of muscle contraction type on CMC

Regarding the type of muscle contraction, CMC is generally lower during isometric contractions compared to other types of contractions. Gwin and Ferris (2012) suggested that voluntary contraction influences the frequency of corticospinal oscillations. Specifically, isotonic contractions shift corticospinal oscillatory activity toward the gamma frequency range, whereas isometric contractions tend to favor oscillations in the beta range (Gwin and Ferris, 2012). Moreover, a study showed that eccentric contractions enhance afferent transmission by increasing muscle spindle activity, thereby imposing greater integrative demands on neural processing (Burke et al., 1978). According to Masakado and Nielsen (2008), the higher coherence during quasi-isotonic contractions than isometric contractions may indicate a greater need for precise control of joint position. Synchronous neuronal firing could promote more efficient signal coupling. Therefore, common oscillatory activity might play a more prominent role in quasi-isotonic than in isometric contractions to coordinate functionally related cortical areas.

### 4.5 Factors that did not consistently influence or lacked evidence of effect

Factors such as training, fatigue, contraction level, postural tasks, walking tasks, and different tasks do not appear to consistently influence CMC. However, the high heterogeneity across studies, particularly in the ‘motor task’ category, limits possibility to gather and interpret studies with similar methodologies. In addition, inter-individual variability in CMC is well documented, even among healthy young adults (Hashimoto et al., 2010; Ushiyama et al., 2011). It is important to note that training-related effects on CMC are highly dependent on the specific task demands and training objectives, and differences across studies should therefore not be interpreted as evidence of weak or unreliable training effects. Moreover, the inconsistent findings related to fatigue may reflect between-study differences in muscle groups tested (proximal knee flexors/extensors vs. distal tibialis anterior), fatigue protocol characteristics (maximal-rate dynamic contractions vs. sustained or submaximal isometric tasks), and/or the timing of CMC assessment, all of which may differentially influence corticospinal drive and oscillatory synchronization.

### 4.6 Methodological factors used to measure EEG–EMG and compute CMC

Most studies investigating CMC have utilized EEG to measure brain activity. First, because MEG requires more advanced instrumentation than EEG, making it significantly more costly then less available for research. In addition, while MEG enables faster data acquisition by eliminating the need for scalp electrode placement, it requires complete subject immobility during recording. Conversely, EEG allows for long-term and telemetric recordings, offering greater adaptability (Hämäläinen et al., 1993). One potential advantage of using MEG over EEG is its greater spatial source localization accuracy. However, the superiority of MEG over EEG remains an open debate, as current evidence is drawn from theoretical models and empirical studies, many of which have technical limitations. A notable example of this controversy comes from Cohen et al. (1990), which suggested that MEG offers only a slight improvement over EEG in detecting the sources of brain activity. However, this conclusion has been challenged on methodological grounds by Hari et al. (1991); Williamson (1991). Overall, researchers mostly used EEG to compute CMC, probably because of its lower cost, greater versatility compared to MEG, and the possibility of performing limb movements in different postures.

The rectification of EMG signals for CMC calculation also remains a subject of ongoing debate in the field, with various studies presenting differing viewpoints regarding its impact on the accuracy and interpretation of CMC results. For instance, Halliday et al. (1995) stated that EMG signals should be rectified, as full-wave rectification is known to provide the temporal pattern of grouped firing of motor units. On the other hand, McClelland et al. (2012) demonstrated that rectification is a non-linear process that significantly distorts the frequency content of EMG signals, thereby compromising the validity of coherence analysis. Although this review does not intend to resolve this debate, our results show that most researchers used rectification of EMG signals (82%) to compute EMG. However few studies justified this methodological choice.

Most studies employed the electrode Cz as the EEG channel for coherence analysis. Cz was chosen because CMC is typically strongest at this site, which lies “superficially” over the sensorimotor region of the cortex (Salenius et al., 1997). Most studies have used single-channel analysis, and only a few have applied source localization techniques. This is mainly because using a single EEG channel to compute CMC is simpler and faster in terms of data processing. Source localization improves the spatial resolution of EEG, complementing its strong temporal precision. Estimating the cortical origins of electrical activity, provides a more accurate understanding of the neural mechanisms underlying cognition, behavior, and neurological conditions (Gomez-Tapia et al., 2025). Despite these advantages, the use of source localization has not been widely adopted to compute CMC.

### 4.7 Limitations

This scoping review has several limitations that should be acknowledged. First, the search was limited to articles published in English and French, which may have led to the exclusion of relevant studies in other languages. Second, although multiple databases were used, there is a possibility that some studies were missed. Third, due to the nature of scoping reviews, no formal assessment of methodological quality or risk of bias was conducted, which limits the ability to comment on the strength of the evidence. Nonetheless, formal assessment is not mandatory for scoping review (Tricco et al., 2018). Fourth, the heterogeneity in study methodologies limited the possibility to compare findings across studies. Fifth, the Methodological factors reported for computing CMC may have differed if we had included studies computing CMC of upper limbs. Sixth, the small median sample size across studies (n = 15) may limit statistical power and should be considered when interpreting the results. Last, the categorization of factors was based on the authors’ judgment, which may introduce a degree of subjectivity.

### 4.8 Research implications and future perspectives

This review has several research implications. First, it identifies multiple factors that have been empirically tested as potentially influencing the CMC of the lower limb and trunk muscles. These factors are presented in Figure 2. Second, among these factors, we identified those that consistently affect CMC (i.e., age, athletic status, contraction type, and muscle), thereby improving our understanding of the physiological mechanisms underlying these phenomena. Third, we identified research gaps, namely, factors for which current data remain uncertain due to study heterogeneity or limited research. For example, the “motor tasks” factor encompassed a wide range of tasks (e.g., gait and postural tasks) that differed across the included studies, limiting our ability to interpret the data. Future research should consider using motor tasks that have already been tested to reduce this heterogeneity. Moreover, we noted a lack of research in certain areas. For instance, it is surprising that no study has used brain stimulation techniques to better understand the cortical areas that may contribute to the sensorimotor control of lower-limb and trunk muscles using CMC. Lastly, we highlighted ongoing methodological debates in the CMC field regarding data collection and processing. An expert consensus could help reduce the diversity of techniques used and thereby minimize methodological heterogeneity.

## 5 Conclusion

This scoping review identified several key factors potentially influencing CMC in axial and lower limb muscles, with age, muscle-specific differences, contraction type, and athletic status emerging as the most consistent contributors. However, considerable heterogeneity in experimental design, motor tasks, and analytical approaches across studies limits direct comparisons and the establishment of strong causal relationships. Methodological choices, such as the use of EEG over MEG, EMG rectification, and reliance on single-channel analyses, also impact the interpretation of results and highlight the need for more standardized protocols. Future research employing source localization and longitudinal designs may offer deeper insights into the neural mechanisms underlying CMC and its modulation by individual and task-related factors.
