# Supplementary material for: Factors influencing corticomuscular coherence for axial and lower limb musculature in a healthy population: a scoping review
Source: Front Hum Neurosci. 2026 Mar 11;20:1708259. doi: 10.3389/fnhum.2026.1708259 (PMC13012959; doi:10.3389/fnhum.2026.1708259)
Supplement: Supplementary file 2 [file Table_2.DOCX]

Supplementary material 2: Research strategies

| 1 | ("corticomuscular coherence" or "cortico-muscular coherence" or (("motor cort*" or "cort* motor*" or "sensorimotor cort*" or "frontal lobe*" or "motor area*" or "frontal area*" or "frontal cort*") and coherence)). ti,ab,kf. |
| --- | --- |
| 2 | \|  \| (Sensorimotor Cortex/ or motor cortex/ or Frontal Lobe/) and coherence.ti,ab,kf. \| \| --- \| --- \| |
| 3 | 1 or 2 |
| 4 | (electromyogra* or "electr* myogra*" or emg). ti,ab,kf. |
| 5 | Electromyography/ |
| 6 | 4 or 5 |
| 7 | (electroencephalogra* or "electr* encephalogra*" or eeg or "brain wave*" or brainwave* or alpha* or beta* or gamma* or "mu rhythm" or "cortical synchroniz*" or magnetoencephalogra* or "magnet* encephalogra*" or meg). ti,ab,kf. |
| 8 | Electroencephalography/ or Brain waves/ or Alpha rhythm/ or Beta rhythm/ or Gamma Rhythm/ or Electroencephalography Phase Synchronization/ or Cortical Synchronization/ or Magnetoencephalography/ |
| 9 | 7 or 8 |
| 10 | 3 and 6 and 9 |
